# Complete genome sequence of *Maribacter* sp. strain C-4077, isolated from the cell surface of a symbiotic dinoflagellate of the bivalve *Fragum* sp.

**DOI:** 10.1128/mra.00358-25

**Published:** 2025-08-11

**Authors:** Toshiyuki Takagi, Kako Aoyama

**Affiliations:** 1 Atmosphere and Ocean Research Institute, The University of Tokyo 13143, Kashiwa, Japan; 2 Graduate School of Frontier Sciences, The University of Tokyo 13143, Kashiwa, Japan; The University of Arizona, Tucson, Arizona, USA

**Keywords:** symbiotic dinoflagellate, bivalve, carotenoids, Flavobacteriaceae

## Abstract

A *Maribacter* sp. strain C-4077 was isolated from an endosymbiotic dinoflagellate of a bivalve and the genome was sequenced using a PacBio Sequel IIe system. The genome consists of a circular 4,085,762 bp chromosome and is predicted to harbor 6 rRNA genes, 39 tRNA genes, and 3,473 coding sequences.

## ANNOUNCEMENT

The genus *Maribacter* of the family Flavobacteriaceae was first described by Nedashkovskaya et al. in 2004 (1). Members of the genus *Maribacter* are often isolated from marine environments such as seawater, shallow marine sediments, deep-sea sediments, phytoplankton, and seaweeds (1–7). Notably, *Maribacter* sp. strain C-4077 was isolated from the cell surface of an endosymbiotic dinoflagellate in the family Symbiodiniaceae (genus *Cladocopium*) of a bivalve (7). The dinoflagellate host was originally collected from the bivalve, *Fragum* sp., in Okinawa, Japan. Taxonomic identification using the EzBioCloud database (8) confirmed that the 16S rRNA gene sequence of strain C-4077 shows 100% similarity to that of *M. flavus* KCTC 42508^T^. There is a mutualistic interaction between the dinoflagellate and C-4077 (7). The dinoflagellate secretes an extracellular matrix forming the microalgal phycosphere, which protects bacteria from antibiotics. Meanwhile, C-4077 protects microalgal cells from light stress through carotenoid production. Sequencing the genome of C-4077 provides insights into the molecular basis of mutualistic interactions between marine Flavobacteriaceae and symbiotic dinoflagellates, which are crucial for understanding stress mitigation in coral reef ecosystems. Here, we provide the complete genome sequence of *Maribacter* sp. strain C-4077.

A single colony of C-4077 was purified in 1/10-strength ZoBell’s 2216E agar medium and stored as frozen glycerol stock cultures at −80°C until use. C-4077 was cultivated on marine agar plates at 25°C for 5 days, and then re-cultivated in 1/5-strength ZoBell’s 2216E liquid medium at 25°C for 24 h. Cultured cells were harvested by centrifugation at 3,000 × g for 5 min, and genomic DNA was extracted using a Qiagen Genomic-tip 20/G and Qiagen DNA Buffer Set according to the Qiagen Genomic DNA Handbook (Qiagen, Hilden, Germany). After fragmenting the genomic DNA to 10–20 kb using g-TUBE (Covaris, Woburn, MA, USA), a SMRTbell Express Template Prep Kit v2.0 (Pacific Biosciences [PacBio], Menlo Park, CA, USA) was used for library preparation following the manufacturer’s recommendations. A SMRTBell polymerase complex was obtained using a binding kit 2.2 (PacBio) and sequenced with a PacBio Sequel IIe (PacBio) by Bioengineering Lab. Co., Ltd. (Kanagawa, Japan). The raw-read N50 was 15,568 bp. Default parameters were used for all software unless otherwise specified. Sequence data were imported into SMRT Link v.10.1.0.119528 to obtain high-fidelity (HiFi) reads with quality values >20. Using Filtlong v.0.2.0 (https://github.com/rrwick/Filtlong), HiFi reads of >1,000 bp were selected, which yielded 65,119 reads (parameters: minimum length 1,000 and keep 90%). The resultant reads were used to assemble contigs with Flye v.2.8.3 (9), which yielded a single contig (4,085 kb in length, with 50-fold coverage).

CheckM v.1.2.2 was used to check the quality of the assembled genome sequence (10), with completeness of 99.7% and contamination of 0.5%. Genome annotation was performed using the DDBJ Fast Annotation and Submission Tool (DFAST) (https://dfast.ddbj.nig.ac.jp/) (11). The complete *Maribacter* sp. strain C-4077 genome comprises a circular 4,085,762 bp chromosome and exhibits a G + C content of 42.1%, with 3,473 coding sequences, 6 rRNA genes, and 39 tRNA genes. The circular chromosome was reoriented to start at the *dnaA* gene.

## Data Availability

The assembled genome sequence and annotations for *Maribacter* sp. C-4077 were deposited in DDBJ/EMBL/GenBank under accession number AP038807, and the sequencing data were deposited in the SRA under accession number DRX562185.
